# Higher Noncardia Gastric Cancer Risk Occurs Earlier in Hispanic and Asian Populations

**DOI:** 10.1016/j.gastha.2026.101032

**Published:** 2026-06-10

**Authors:** Chul S. Hyun, Rong Wang

**Affiliations:** 1 Section of Digestive Diseases, Yale School of Medicine, New Haven, Connecticut; 2 Chronic Disease Epidemiology, Yale School of Public Health, New Haven, Connecticut

**Keywords:** Age-Specific Incidence, Asian Ancestry, Hispanic Populations, Noncardia Gastric Cancer, Risk Stratification

## Abstract

**Background and Aims:**

Noncardia gastric cancer (GC) incidence in the United States varies substantially by race and ethnicity, but age-adjusted estimates do not indicate when higher-risk populations reach clinically meaningful levels of disease. We evaluated age-specific incidence across racial/ethnic and Asian ancestry groups to characterize differences in both the magnitude and timing of risk relative to non-Hispanic White (NHW) populations.

**Methods:**

We conducted a population-based analysis of noncardia GC incidence in California and New York from 2010 to 2022. Age-specific rates were estimated for NHW, Hispanic, and non-Hispanic Asian/Pacific Islander populations and for Asian subgroups (Chinese, Japanese, Korean, and Vietnamese). Rate ratios were calculated relative to NHW populations, and the age at which each group reached the incidence observed among NHW adults aged 60 to 69 years was identified.

**Results:**

Incidence increased with age and remained higher among Hispanic and Asian populations than among NHW populations. Substantial heterogeneity was observed across Asian ancestries, with rate ratios ranging from 2.83 to 5.98 at ages 40 to 49 and reaching 12.04 among Korean individuals at ages 60 to 69. Incidence levels observed among NHW adults aged 60 to 69 were reached up to 2 decades earlier in several Asian populations.

**Conclusion:**

Age-specific incidence of noncardia GC is higher among Hispanic and Asian populations than among NHW populations, with marked heterogeneity across Asian ancestries. These findings indicate a shift in the age distribution of risk, with clinically meaningful incidence occurring earlier in higher-risk populations. This age-specific pattern provides population-based evidence to inform risk assessment and consideration of earlier, risk-based prevention strategies.

## Introduction

Gastric cancer (GC) is the fifth most common cancer and the fourth leading cause of cancer death worldwide.^1^^,^^2^ In the United States, incidence varies substantially across racial and ethnic groups.^2^, ^3^, ^4^ Noncardia gastric adenocarcinoma accounts for approximately two-thirds of cases and is strongly associated with *Helicobacter pylori* infection, as well as other modifiable environmental and lifestyle factors, including dietary patterns, smoking, and alcohol use.^5^, ^6^, ^7^ Noncardia GC is frequently diagnosed at an advanced stage and is associated with poor outcomes.^8^^,^^9^ Population-based studies report an overall 5-year survival of approximately 26%, with survival as low as 2%–6% for distant-stage disease, underscoring the importance of prevention and early detection.^9^

Substantial racial and ethnic disparities in GC incidence and its precursor lesions have been consistently documented. Asian and Hispanic populations experience a higher burden than non-Hispanic White (NHW) populations,^4^^,^^9^, ^10^, ^11^ reflecting differences in early-life exposures, environmental and behavioral risk factors, and underlying susceptibility. A population-based analysis by Shah and colleagues using California registry data demonstrated higher noncardia GC incidence among Asian populations compared with NHW among adults aged 50 years and older.^4^ However, prior studies have largely relied on age-restricted analyses or summary age-adjusted rates, which provide limited information on the age-specific distribution of risk.

Although age-adjusted rates describe overall population burden, they do not indicate when higher-risk populations reach clinically meaningful levels of disease. Clinical evaluation and prevention decisions in the United States are typically guided by age-based thresholds that reflect risk patterns in the general population.^12^^,^^13^ For conditions with strong age gradients such as noncardia GC, populations with similar age-adjusted rates may reach comparable levels of risk at substantially different ages. Identifying the age at which higher-risk populations reach risk levels observed in the general population provides a more clinically interpretable framework than overall or age-restricted incidence estimates.

Contemporary cancer registry data linked to population denominators allow stable estimation of age-specific incidence in regions with large and diverse Asian populations. California and New York together account for a substantial proportion of the US Asian population and provide sufficient case volume to examine multiple Asian ancestry groups.^14^^,^^15^ Data extending through 2022 further allow assessment of current patterns of disease burden.

The objective of this study was to evaluate age-specific incidence of noncardia GC in California and New York from 2010 through 2022. We estimated incidence across successive adult age groups and compared rates among NHW, Hispanic, and non-Hispanic Asian or Pacific Islander (NHAPI) populations, as well as among a combined high-risk Asian group (Chinese, Japanese, Korean, and Vietnamese). We further examined heterogeneity across these 4 Asian ancestries and evaluated the age at which these populations reached incidence levels observed among NHW adults, providing a clinically interpretable framework for comparing the timing of risk across populations.

## Methods

### Data Source and Study Population

We conducted a population-based descriptive analysis using data from the Surveillance, Epidemiology, and End Results (SEER) Program.^16^ Incident GC cases diagnosed between 2010 and 2022 were identified from SEER registries in California and New York, 2 states with large and diverse Asian populations and sufficient case volume for stable subgroup-specific estimates.^14^^,^^15^

Gastric adenocarcinoma cases were identified using International Classification of Diseases for Oncology, Third Edition topography codes C16.0–C16.9 and relevant adenocarcinoma histology codes. Tumors were classified by anatomic subsite as cardia (C16.0) or noncardia (C16.1–C16.9), and the present analysis was restricted to noncardia GC.

Race and ethnicity were categorized as NHW, Hispanic (all races), and NHAPI. Asian ancestry was further classified using the SEER Race recode (with detailed Asian and Native Hawaiian/Pacific Islander categories). Four Asian subgroups—Chinese, Japanese, Korean, and Vietnamese—were included a priori based on sufficient case volume and prior studies demonstrating elevated GC incidence in these populations.^14^^,^^15^

The combined California–New York dataset included 35,375 noncardia GC cases, including 8125 among NHAPI individuals and 11,703 among Hispanic individuals. Within Asian subgroups, case counts included approximately 3294 Chinese, 1667 Korean, 775 Vietnamese, and 630 Japanese cases.

### Population Denominators

Population denominators for overall race/ethnicity categories (NHW, NHAPI, and Hispanic) were obtained from SEER. For detailed Asian subgroups, population estimates were derived from the American Community Survey (ACS) Public Use Microdata Sample for California and New York.^17^

Asian subgroup populations were defined using the detailed race variable (RAC2P) to ensure mutually exclusive classification and consistency with cancer surveillance practices. Population estimates for each age–race stratum were calculated using ACS person-level weights. Sensitivity analyses using ancestry-based denominators derived from the first-reported ancestry variable (ANC1P) produced similar incidence patterns; therefore, results are presented using RAC2P-defined populations. Although SEER and ACS race classifications are not derived from identical sources, both use standardized federal race categories, consistent with prior population-based studies.

### Incidence Estimation

Age-specific incidence rates were calculated for the following age groups: 0 to 39, 40 to 49, 50 to 59, 60 to 69, and ≥70 years. Overall incidence rates were age-adjusted to the 2000 US standard population.^18^

To improve stability for smaller populations, incidence rates were calculated using multiyear aggregated data across the 2010 to 2022 study period. Estimates based on small case counts were interpreted cautiously in accordance with SEER reporting guidelines.

### Statistical Analysis

Rate ratios (RRs) were calculated for Hispanic and Asian populations relative to NHW populations within each age group. Ninety-five percent confidence intervals were estimated using the log-RR method.

Overall incidence rates for SEER race/ethnicity categories were generated using SEER∗Stat (version 9.0.42.2) and SAS (version 9.4; SAS Institute, Cary, North Carolina). For detailed Asian subgroups, incidence rates were calculated by dividing SEER case counts by ACS-derived population denominators, with standard errors estimated using Poisson methods in SAS.

All analyses were descriptive and were not intended to estimate causal associations. No statistical modeling or adjustment beyond age standardization was performed. Because the study used deidentified, publicly available data, institutional review board approval was not required.

## Results

### Overall Incidence by Race/Ethnicity

Age-adjusted incidence of noncardia GC differed substantially across racial and ethnic groups (Table 1). Overall incidence was 2.21 per 100,000 among NHW populations, compared with 7.55 per 100,000 among NHAPI populations and 7.01 per 100,000 among Hispanic populations. Overall incidence was 3.41 times higher among NHAPI populations and 3.17 times higher among Hispanic populations compared with NHW populations.

**Table 1:** Overall Age-Adjusted Incidence of Noncardia Gastric Cancer, 2010–2022

Group	Cases	Rate per 100,000 (95% CI)	Rate ratio (95% CI)
Non-Hispanic White	11,105	2.21 (2.17–2.25)	Reference
Non-Hispanic Asian/Pacific Islander	8125	7.55 (7.38–7.71)	3.41 (3.31–3.51)
Hispanic	11,703	7.01 (6.87–7.14)	3.17 (3.08–3.25)

CI, confidence interval.

Rates age-adjusted to the 2000 US standard population.

### Age-Specific Incidence by Race/Ethnicity and Asian Ancestry

**Figure 1:**
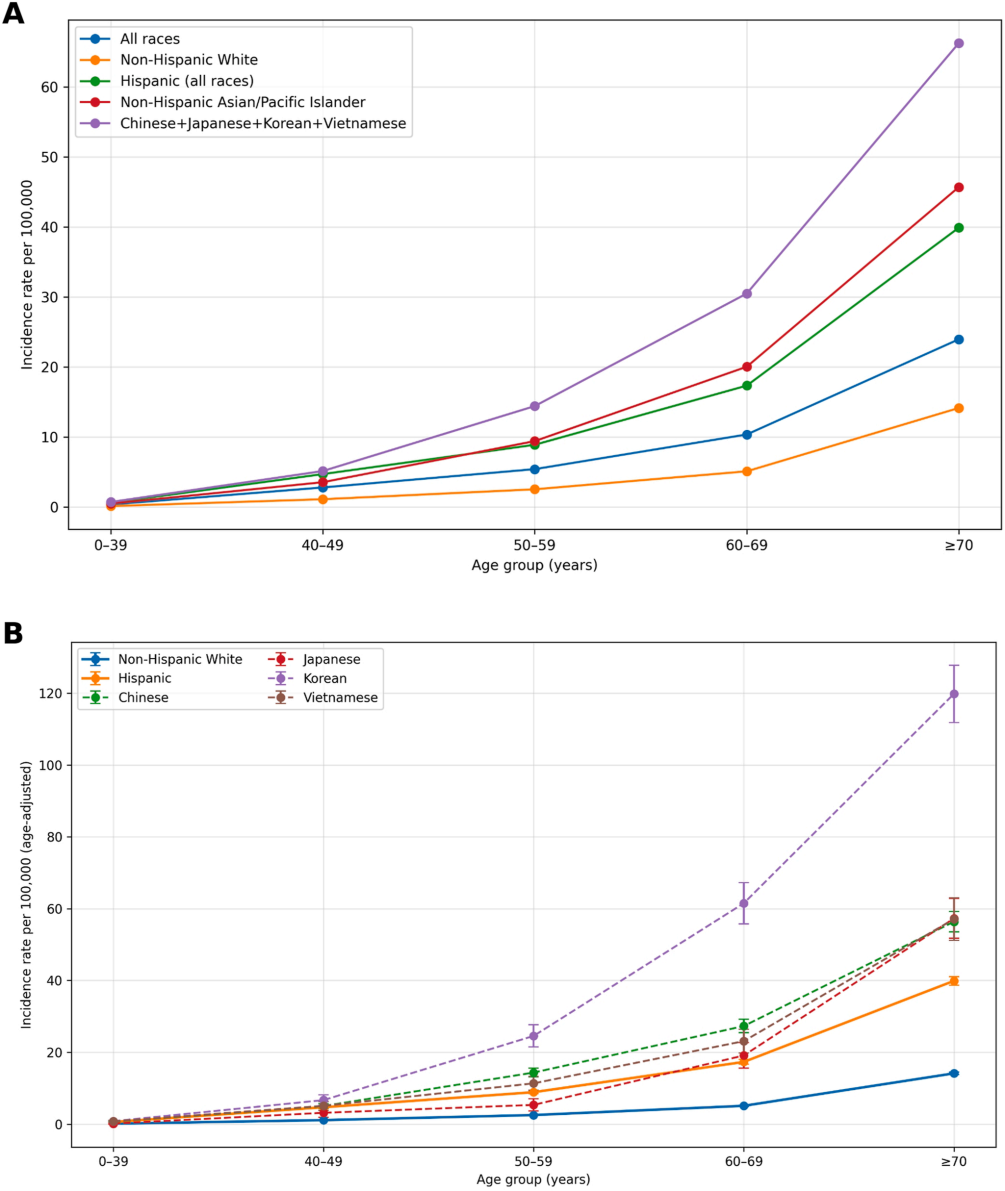
Age-specific noncardia gastric cancer incidence rates in California and New York, 2010 to 2022, stratified by race/ethnicity and ancestry. (A) Incidence rates by major racial/ethnic groups, including all races, non-Hispanic White, Hispanic, non-Hispanic Asian/Pacific Islander, and combined Chinese, Japanese, Korean, and Vietnamese populations. (B) Incidence rates with 95% confidence intervals stratified by individual ancestry groups, including Chinese, Japanese, Korean, and Vietnamese populations, compared with Hispanic and non-Hispanic White populations.

Age-specific incidence increased with age across all racial and ethnic groups and remained higher than that observed among NHW populations (Figure 1A). Among NHW populations, incidence rose from 1.12 per 100,000 at ages 40 to 49 to 14.15 per 100,000 at ages ≥70. Rates were higher among Hispanic populations, increasing from 4.71 per 100,000 at ages 40 to 49 to 39.89 per 100,000 at ages ≥70. Among NHAPI populations, incidence increased from 3.55 per 100,000 at ages 40 to 49 to 45.69 per 100,000 at ages ≥70. When analyses were limited to the combined Chinese, Japanese, Korean, and Vietnamese populations, incidence ranged from 5.15 per 100,000 at ages 40 to 49 to 66.25 per 100,000 at ages ≥70 (Figure 1A).

Within Asian populations, age-specific incidence varied substantially by ancestry (Figure 1B). At ages 40 to 49, incidence ranged from 3.17 per 100,000 among Japanese individuals to 6.69 per 100,000 among Korean individuals. Differences across groups were greater at older ages. At ages 50 to 59, rates were 5.37 per 100,000 for Japanese, 11.37 for Vietnamese, 14.37 for Chinese, and 24.58 for Korean populations. At ages 60 to 69, incidence increased to 19.13 per 100,000 among Japanese, 23.12 among Vietnamese, 27.32 among Chinese, and 61.51 among Korean individuals. Among adults aged ≥70 years, incidence ranged from 56.38 to 57.28 per 100,000 for Chinese, Japanese, and Vietnamese populations and reached 119.80 per 100,000 among Korean individuals. Across all age groups, incidence among Hispanic populations and each Asian subgroup exceeded that observed among NHW populations.

### Rate Ratios by Race/Ethnicity and Ancestry

Age-specific incidence rates for Hispanic and Asian populations were higher than those for NHW populations across all age groups (Table 2). Among Hispanic populations, incidence was approximately 4.2 times higher at ages 40 to 49, 3.5 times higher at ages 50 to 59, 3.4 times higher at ages 60 to 69, and 2.8 times higher at ages ≥70.

**Table 2:** Age-Specific Incidence of Noncardia Gastric Cancer by Ancestry, California and New York

Age group	NH White rate (95% CI)	Hispanic rate (95% CI)	RR	Chinese rate (95% CI)	RR	Japanese rate (95% CI)	RR	Korean rate (95% CI)	RR	Vietnamese rate (95% CI)	RR
0–39	0.14 (0.12–0.16)	0.61 (0.57–0.66)	4.36	0.74 (0.60–0.88)	5.27	0.17 (0.00–0.34)	1.23	0.84 (0.57–1.11)	5.98	0.80 (0.53–1.07)	5.73
40–49	1.12 (1.02–1.22)	4.71 (4.47–4.95)	4.21	5.05 (4.35–5.75)	4.51	3.17 (1.87–4.47)	2.83	6.69 (5.26–8.13)	5.98	5.11 (3.87–6.34)	4.56
50–59	2.54 (2.40–2.68)	8.90 (8.54–9.28)	3.50	14.37 (13.17–15.56)	5.66	5.37 (3.67–7.06)	2.11	24.58 (21.50–27.65)	9.67	11.37 (9.39–13.34)	4.47
60–69	5.11 (4.90–5.32)	17.34 (16.68–18.03)	3.39	27.32 (25.47–29.17)	5.35	19.13 (15.57–22.69)	3.74	61.51 (55.77–67.26)	12.04	23.12 (19.80–26.44)	4.53
≥70	14.15 (13.81–14.50)	39.89 (38.72–41.08)	2.82	56.38 (53.58–59.18)	3.98	57.28 (51.74–62.81)	4.05	119.80 (111.80–127.80)	8.47	57.10 (51.17–63.03)	4.03

CI, confidence interval.

Population denominators were derived from American Community Survey detailed race (RAC2P). Rates are age-specific and expressed per 100,000 person-years. Rate ratios (RRs) represent descriptive comparisons relative to non-Hispanic White populations within the same age group, which served as the reference category.

Among Asian subgroups, RRs varied by ancestry and age. For example, at ages 40 to 49, RRs ranged from 2.83 for Japanese to 5.98 for Korean populations, increasing at ages 50 to 59 to 2.11 and 9.67, respectively. At ages 60 to 69, RRs ranged from 3.74 for Japanese to 12.04 for Korean populations, while at ages ≥70, RRs ranged from 3.98 to 4.05 for Chinese, Japanese, and Vietnamese populations and were 8.47 for Korean populations (Table 2).

### Risk-Equivalent Age Relative to Non-Hispanic White Populations

**Figure 2:**
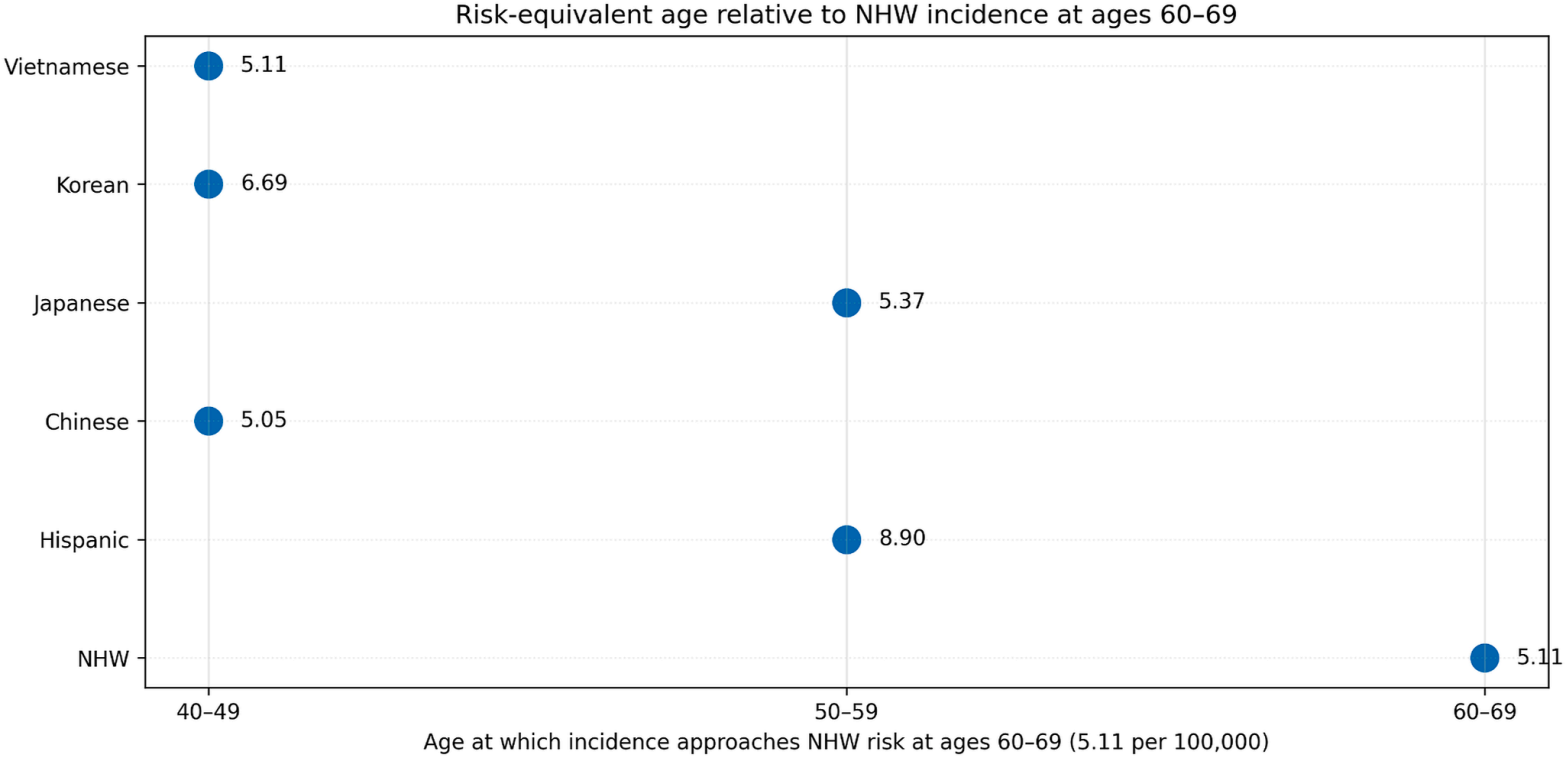
Age at which noncardia gastric cancer incidence approaches the non-Hispanic White risk at ages 60 to 69 years (California and New York, 2010–2022). Age group at which noncardia gastric cancer incidence in each racial/ethnic group approaches the incidence observed among non-Hispanic White (NHW) individuals aged 60 to 69 years (5.11 per 100,000). Points indicate the earliest age category meeting or closely approximating this benchmark; labels show age-specific incidence rates (per 100,000). Korean, Vietnamese, and Chinese populations reached comparable levels at ages 40 to 49, whereas Japanese and Hispanic populations did so at ages 50 to 59. Rates are age-specific and expressed per 100,000 person-years based on California and New York cancer registry data, 2010 to 2022.

We examined the age at which noncardia GC incidence in each group approached the incidence observed among NHW individuals aged 60 to 69 years (5.11 per 100,000) (Figure 2). Korean and Vietnamese populations reached comparable incidence levels by ages 40 to 49 (6.69 and 5.11 per 100,000, respectively). Chinese, Japanese, and Hispanic populations reached similar levels in ages 50 to 59 (5.05, 5.37, and 8.90 per 100,000, respectively). Incidence levels observed among NHW individuals aged 60 to 69 were reached in the 40 to 49 or 50 to 59 age groups in several higher-risk populations.

## Discussion

### Epidemiologic Findings

In this population-based analysis of noncardia GC in California and New York from 2010 through 2022, incidence increased with age across all racial and ethnic groups and varied substantially by race/ethnicity and Asian ancestry. Hispanic and NHAPI populations had higher incidence than NHW populations at all ages (Figure 1A). Overall incidence was approximately threefold higher among Asian and Hispanic populations compared with NHW (Table 1). Within Asian populations, marked heterogeneity was observed (Figure 1B). Korean individuals had the highest age-specific incidence, reaching 119.8 per 100,000 at ages ≥70, while Chinese, Japanese, and Vietnamese populations also had consistently elevated rates compared with NHW.

These findings are consistent with prior population-based studies demonstrating racial and ethnic disparities in noncardia GC incidence in the United States. In particular, the analysis by Shah and colleagues, based on California registry data, reported higher incidence among Asian populations compared with NHW populations among adults aged 50 years and older.^4^ The present study extends this work by incorporating data from both California and New York and by using contemporary data through 2022. In addition, incidence was evaluated across successive adult age groups, allowing characterization of the timing and distribution of risk across the adult life course.

Importantly, this analysis highlights differences not only in the magnitude of risk across populations but also in the timing of risk emergence, with several higher-risk groups reaching clinically meaningful incidence levels at younger ages than NHW populations. These findings also relate to observations of earlier age at presentation in certain populations^19^ and underscore the importance of understanding age-specific patterns of risk. Although earlier age at presentation has been described in the context of early-onset GC, the patterns observed here reflect broader differences in the age distribution of incidence rather than a phenomenon limited to early-onset disease.

Age-specific estimates provide clinically interpretable information not reflected in age-adjusted rates. Elevated incidence among Asian populations was evident beginning at ages 40 to 49 and increased with advancing age (Figure 1A). Relative differences compared with NHW were observed across all age groups (Table 1), with RRs as high as 12.04 among Korean individuals at ages 60 to 69. These findings highlight substantial variation in both the magnitude and timing of risk across Asian ancestries.

The focus on noncardia GC is clinically relevant. Noncardia tumors represent the predominant form of GC in high-risk populations and are strongly associated with *H pylori* infection and other environmental exposures acquired earlier in life.^5^, ^6^, ^7^ Because outcomes remain poor and the disease is potentially preventable, defining age-specific risk patterns is important for prevention planning.

### Clinical Implications

From a clinical perspective, the absence of US population-based screening recommendations for GC has led to reliance on symptom-based evaluation rather than quantitative risk thresholds.^20^ Current American College of Gastroenterology and Canadian Association of Gastroenterology guidelines for the management of dyspepsia recommend upper endoscopy at age ≥60 years for evaluation of upper gastrointestinal symptoms, without race- or ethnicity-specific risk stratification.^21^

In this context, the present analysis shows that age-specific incidence levels observed among NHW adults aged 60 to 69 years were reached or closely approached up to 2 decades earlier in several Asian populations (Figure 2). These findings indicate that levels of GC risk typically encountered at older ages in the general population may occur earlier in certain higher-risk groups, reflecting a shift in the age distribution of risk.

This pattern likely reflects the combined influence of established risk factors, including *H pylori* infection, genetic susceptibility, environmental and lifestyle exposures, migration-related factors, and differences in socioeconomic conditions and access to care. Importantly, these findings are derived from population-based datasets in California and New York, which include substantial representation of higher-risk populations and allow stable estimation of age-specific incidence patterns.

These findings have implications for earlier risk assessment and consideration of risk-based evaluation strategies in higher-risk populations, particularly those with elevated risk related to early-life exposures prior to migration.^22^ Together, the age-specific patterns shown in Figures 1 and 2 and the relative differences summarized in Table 1 provide a clinically interpretable framework for understanding the timing and magnitude of GC risk across diverse populations in the United States and may help inform risk stratification and targeted prevention strategies.

### Limitations and Strengths

Registry data do not include individual-level information on *H pylori* status, nativity, duration of residence in the United States, or socioeconomic factors, all of which may influence GC risk and contribute to observed differences across populations. More granular stratification, including by Hispanic subgroups or immigrant generation, would further inform underlying mechanisms but is not feasible within population-based registry datasets.

The present analysis leverages population-based data from California and New York, 2 of the largest and most diverse populations in the United States, providing sufficient statistical power to generate stable age-specific incidence estimates across major racial and ethnic groups. Although not nationally comprehensive, these states include substantial representation of populations at highest risk and allow stable subgroup-specific estimation and are widely used for population-based epidemiologic analyses in the United States.

Within this context, the primary aim of this study is to characterize population-level patterns in the timing and distribution of GC risk rather than to define underlying etiologic mechanisms. Further studies incorporating more detailed individual-level risk factors are needed to better understand the drivers of these observed patterns.

Strengths include the use of contemporary data through 2022, a large number of cases across multiple racial/ethnic groups, and stable subgroup-specific estimates. By focusing on age-specific incidence and clinically interpretable comparisons, this study provides a population-based framework for evaluating GC risk across diverse populations.

## Conclusion

Age-specific incidence of noncardia GC is higher among Hispanic and Asian populations than among NHW populations beginning in midlife, with marked heterogeneity across Asian ancestries and the highest rates observed among Korean individuals. In several higher-risk populations, incidence levels observed among NHW at older ages occur up to 2 decades earlier. These contemporary population-based estimates provide clinically interpretable information on the timing and magnitude of risk not captured by aggregated or age-adjusted measures and may inform risk assessment and targeted prevention and early detection strategies.
